# Cinnamaldehyde alleviates doxorubicin-induced cardiotoxicity by decreasing oxidative stress and ferroptosis in cardiomyocytes

**DOI:** 10.1371/journal.pone.0292124

**Published:** 2023-10-12

**Authors:** Meijiao Mao, Wang Zheng, Bin Deng, Youhua Wang, Duan Zhou, Lin Shen, Wankang Niku, Na Zhang

**Affiliations:** Department of Cardiology, Longhua Hospital, ShangHai University of Traditional Chinese Medicine, Shanghai, China; National Institutes of Health, UNITED STATES

## Abstract

Although doxorubicin (DOX) is an efficient chemotherapeutic drug for human tumors, severe cardiotoxicity restricts its clinical use. Cinnamaldehyde (CA), a bioactive component isolated from Cinnamonum cassia, possesses potent anti-oxidative and anti-apoptotic potentials. The major aim of this study was to evaluate the protective role of CA against DOX-induced cardiotoxicity. To this end, cardiomyocyte injury models were developed using DOX-treated H9c2 cells and DOX-treated rats, respectively. Herein, we found that CA treatment increased cardiomyocyte viability and attenuated DOX-induced cardiomyocyte death *in vitro*. CA further protected rats against DOX-induced cardiotoxicity, as indicated by elevated creatine kinase (CK) and lactate dehydrogenase (LDH) levels, myocardium injury, and myocardial fibrosis. CA alleviated DOX-induced myocardial oxidative stress by regulating reactive oxygen species (ROS), malondialdehyde (MDA), superoxide dismutase (SOD), and glutathione (GSH) levels. Mechanistically, CA markedly accelerated nuclear translocation of nuclear erythroid factor 2-related factor 2 (Nrf2) and increased heme oxygenase-1 (HO-1) expression. Consequently, CA decreased DOX-induced cardiomyocyte ferroptosis, while Erastin (a ferroptosis agonist) treatment destroyed the effect of CA on increasing cardiomyocyte viability. Taken together, the current results demonstrate that CA alleviates DOX-induced cardiotoxicity, providing a promising opportunity to increase the clinical application of DOX.

## Introduction

Despite its proven efficacy in treating multiple types of tumors, the clinical application of DOX is restricted by its severe cardiotoxicity [1, 2]. Emerging evidence has demonstrated that DOX-induced myocardial damage is closely correlated with mitochondrial impairment [3], intracellular calcium homeostasis [4, 5], cardiomyocyte apoptosis [6], ferroptosis [7, 8], and oxidative stress [9, 10].

Oxidative stress is considered a vital cause of DOX-triggered myocardial damage [11]. Semiquinone, derived from DOX via one-electron reduction, can autoxidize by transferring an unpaired electron to O2 and thus generate a superoxide anions free radical (O2-) [11, 12]. O2- results in DNA damage, mitochondrial impairment, and subsequent cardiomyocyte injury [11]. Oxidative stress raises cardiomyocyte Ca^2+^ levels by influencing the sarcolemmal membrane to facilitate Ca^2+^ entry, causing Ca^2+^-handling abnormalities [13, 14]. Oxidative stress also exerts crucial effects on organ damage by triggering ferroptosis [15]. Nrf2 is a critical antioxidant transcription factor [16] and regulates the expression of multiple genes in the process of ferroptosis, such as ferritin heavy chain 1 (FTH1) [17], glutathione peroxide 4 (GPX4) [18, 19], and solute carrier family 7 member 11 (SLC7A11) [20, 21]. Therefore, oxidative stress may be a central event during DOX-induced myocardial injury by triggering apoptosis [22], ferroptosis [23], and pyroptosis [24].

Ferroptosis is a recently identified iron-driven cell death characterized by elevated lipid peroxidation, ROS overload, and plasma membrane rupture [8]. Several genes involved in ferroptosis have been identified, such as NADPH oxidase 1 (NOX1), cyclooxygenase-2 (COX-2), ACSL4, PTGS2, GPX4, FTH1, and SLC7A11 [25]. Ferroptosis is involved in DOX-induced cardiotoxicity [26]. Fang et al. demonstrated that ferrostatin-1 (Fer-1), a potent ferroptosis inhibitor, alleviates DOX-induced mortality, whereas pharmacological intervention of apoptosis, necroptosis, or autophagy could not significantly improve survival in DOX-treated mice [26].

CA, a bioactive component isolated from Cinnamonum cassia, possesses potent anti-oxidative [27, 28], anti-inflammatory [29], and anti-tumor capabilities [30]. Huang et al. demonstrated that CA increases phase II detoxifying enzyme expression by activating Nrf2 in hepatocellular carcinoma cells [27]. Kim et al. reported that CA alleviates dental pulp cell oxidative stress via activating the Nrf2/HO-1 pathway [28]. In the study, the biological effect of CA on alleviating DOX-induced cardiotoxicity was evaluated *in vitro* and *in vivo*.

## Materials and methods

### Cell culture and treatment

H9c2 cells were obtained from the Cell Bank of the Chinese Academy of Sciences (Shanghai, China) and cultivated in DMEM (GIBCO, NY, USA) containing 10% FBS (GIBCO) in a humidified CO2 incubator at 37 °C. H9c2 cells were seeded into 96-well plates (5000 cells per well) or 6-well plates (2 × 10^6^ cells per well) overnight and then treated with DOX (0.5, 1, 2, 4, 8, and 10 μM) for 24 h.

### Rat model of DOX-induced myocardial injury

The protocol was performed with the approval of the Experimental Animal Committee of Longhua Hospital Affiliated to Shanghai University of Traditional Chinese Medicine (No. PZSHUTCM210312009) according to ARRIVE guidelines [31] to decrease animals suffering. Male Sprague-Dawley rats (approximately 8 weeks old) were obtained from the Shanghai Model Organisms Center, Inc. (China) and maintained in specific pathogen-free facilities (temperature: 21–24°C) with water and food ad libitum. In the DOX group, rats (n = 5) were injected intraperitoneally (i.p.) with DOX at doses of 15 mg/kg (DOX was dissolved in 0.9% saline) as previously described [32]. In the CA treatment group, after treatment with 15 mg/kg of DOX, rats (n = 5) were additionally gavaged with CA at a dose of 50 mg/kg for 6 weeks, as previously described [33, 34] and in our preliminary experiments. In the control group (n = 5), rats were injected i.p. with 0.9% saline. Subsequently, rats were euthanized by inhalation of 2% isoflurane to collect blood and heart tissues for subsequent analysis.

### Cell viability

The CCK-8 reagent (Abcam, CA, USA) was applied to measure H9c2 cell viability. Briefly, H9c2 cells were cultured in 96-well plates (5×10^3^ cells per well) and treated with DOX (0.5, 1, 2, 4, 8, and 10 μM) for 24 h in the presence or absence of CA (0, 20, 40, 80, 100, 160, and 200 μM), Fer-1 (1 μM), or Erastin (10 μM). After that, cells were incubated with CCK-8 (10 μL) for 60 min in a humidified CO2 incubator, and then absorbance at 450 nm was read with a DR-200Bc microplate reader (BIOBASE, Shangdong, China).

### TUNEL assay

TUNEL was used to assess H9c2 cell death. In brief, cells were seeded into 12-well plates and then treated with 4 μM of DOX and 100 μM of CA. After treatment for 24 h, cells were fixed with 4% PFA and stained with TUNEL (Beyotime, Shanghai, China) for 60 min. The fluorescence signal was captured with a XSPY-3201LED fluorescent microscope (CSOIF, Shanghai, China).

### Serum biochemical indexes

Several serum biochemical indexes, including CK and LDH, were assessed using commercial kits (CK: ab155901, Abcam, CA, USA; LDH: ab102526, Abcam) in line with the manufacturer’s specifications.

### Intracellular ROS assay

H9c2 cells cultured in 12-well plates (0.1×10^5^ cells / well) were treated with 4 μM of DOX and 100 μM of CA for 24 h. After washing thrice in PBS, cells were incubated with 10 μM of DCFH-DA (MedChem Express, NJ, USA) for 20 min in the dark. The fluorescence signal was captured with a XSPY-3201LED fluorescent microscope (CSOIF, Shanghai, China).

### Assessment of MDA, SOD, GSH, and GSH-Px

Cardiac samples were homogenized in RIPA buffer on ice, and then the supernatant was collected from tissue lysates via centrifuging at 3000 rpm for 15 min at 4°C. MDA, SOD, GSH, and GSH-Px levels in the supernatant were assessed using commercial kits in line with the manufacturer’s specifications.

### Hematoxylin-eosin (HE) and Masson staining

Cardiac tissues were fixed with 4% PFA, embedded in paraffin, serially sectioned, and stained with HE and Masson (Beyotime). Images were examined using an N-800M light microscope (OLABO, Shandong, China).

### Quantitative real-time PCR (qRT-PCR)

Total RNA was collected with TRIzol (Beyotime), and first-strand cDNA was synthesized with M-MLV reverse transcriptase (Sigma-Aldrich, MO, USA) and Oligo (dT)18 primers. qRT-PCR was performed with BeyoFast^™^ SYBR Green qPCR Mix (Beyotime) on a StepOnePlus real-time PCR system (Thermo Fisher Scientific). The temperature procedure is: 10 min at 95°C followed by 35 cycles of 95°C for 25 s and 58°C for 10 s. β-actin was applied as a reference gene. The 2^(-ΔΔCT)^ method was applied to calculate the relative mRNA level [35]. All primers were shown in S1 Table.

### Western blot

Total protein was collected from tissues or cells with RIPA buffer (Thermo Fisher Scientific). Nuclear and cytoplasmic proteins were isolated from cardiac tissues or H9c2 cells using a NE-PER^™^ reagent (Thermo Fisher Scientific) according to the manufacturer’s protocol. Equal amounts of total protein (approximately 30 μg) were separated using 10% SDS-PAGE and transferred to PVDF membranes (Merck, MA, USA). After blocking with 5% no-fat milk, membranes were incubated with antibodies against Nrf2 (1:1000, ab92946, Abcam), HO-1 (1:4000, ab68477), Gpx4 (1:3500, ab125066), Acsl4 (1:10000, ab155282), Ptgs2 (1:3000, ab179800), β-actin (1:4000, ab8226), and Lamin B1 (1:800, ab229025) overnight at 4°C. Then membranes were incubated with HRP-labeled secondary antibodies after washing three times in TBST. Immunoblot was observed using an enhanced chemiluminescence assay (Beyotime).

### Statistics

The data were shown as the mean ± SD from three separate experiments. GraphPad Prism 7.0 (CA, USA) was used to compare the difference between two groups using the student’s t-test or among multiple groups using a one-way ANOVA followed by the Scheffé test. The difference was statistically significant when p < 0.05.

## Results

### CA alleviated DOX-induced myocardial injury

**Fig 1:**
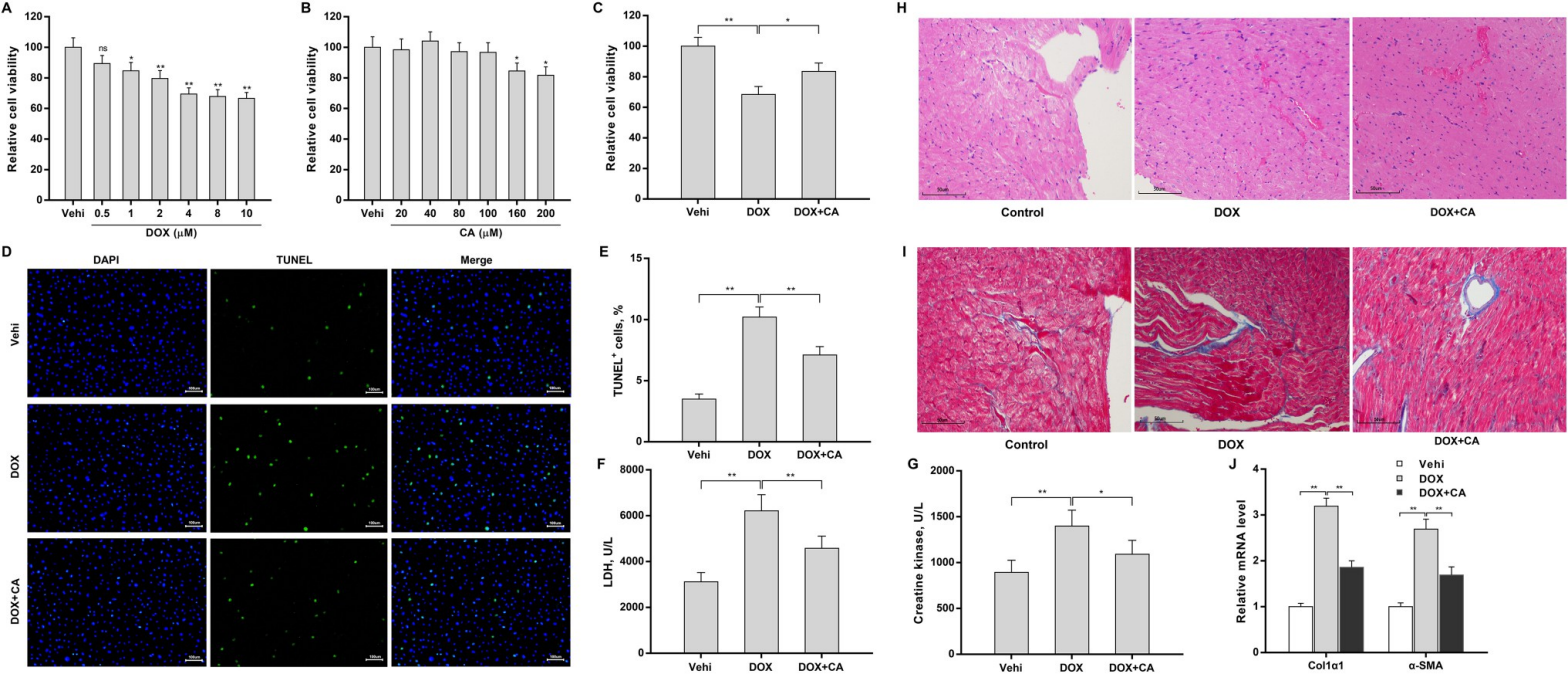
CA alleviated DOX-induced myocardial injury *in vitro* and *in vivo*. H9c2 cells were treated with different doses of DOX (A) or CA (B), and then cell viability was measured using CCK8 after 24 h (n = 3). (C) H9c2 cells were treated with 4 μM of DOX and 100 μM of CA for 24 h, and cell viability was measured using CCK8 after 24 h (n = 3). (D and E) H9c2 cells were treated with 4 μM of DOX and 100 μM of CA for 24 h, and cell death was measured using TUNEL (n = 3). Rats were treated with 15 mg/kg of DOX with or without CA (50 mg/kg, 6 weeks), and serum CK (F) and LDH (G) levels were assessed using commercial kits (n = 5). (H) HE staining was used to assess myocardial injury in rats treated with DOX and CA (n = 5). (I) Masson staining was used to assess myocardial fibrosis in rats treated with DOX and CA (n = 5). qRT-PCR was performed to assess Col1α1 and α-SMA (J) mRNA levels in rats treated with DOX and CA (n = 5). *p<0.05, **p<0.01.

Consistent with previous studies [11, 36], DOX exhibited a dose-dependent cytotoxic effect on H9c2 cells (Fig 1A). Based on the results, 4 μM of DOX was applied to treat H9c2 cells in subsequent experiments. The cytotoxic effects of CA on H9c2 cells were also assessed using the CCK-8 assay. Fig 1B showed that CA did not exhibit a significant cytotoxic effect on H9c2 cells up to 100 μM. Therefore, 100 μM of CA was applied to treat H9c2 cells in subsequent experiments. The protective role of CA against DOX-induced cardiotoxicity was next assessed. As shown in Fig 1C, CA significantly reversed the effect of DOX on inhibiting H9c2 cell viability. CA also alleviated DOX-induced H9c2 cell death (Fig 1D and 1E). Furthermore, DOX treatment increased serum CK and LDH levels in rats, whereas these effects were reversed by CA (Fig 1F and 1G). The results from HE staining showed that DOX resulted in an obvious cardiomyocyte injury, as suggested by misaligned muscle fibers and widened intercellular spaces, whereas these effects were blocked by CA (Fig 1H). Masson staining revealed that DOX increased collagen fibers in the myocardial interstitium, and their distribution was chaotic, whereas myocardial fibrosis was obviously improved and tended to normalize after CA treatment (Fig 1I). CA also decreased DOX-induced Col1α1 and α-SMA expression in heart tissues (Fig 1J). These results demonstrate that DOX exhibits serious cardiotoxicity *in vitro* and *in vivo*, which can be reversed by CA.

### CA alleviated DOX-induced myocardial oxidative stress

**Fig 2:**
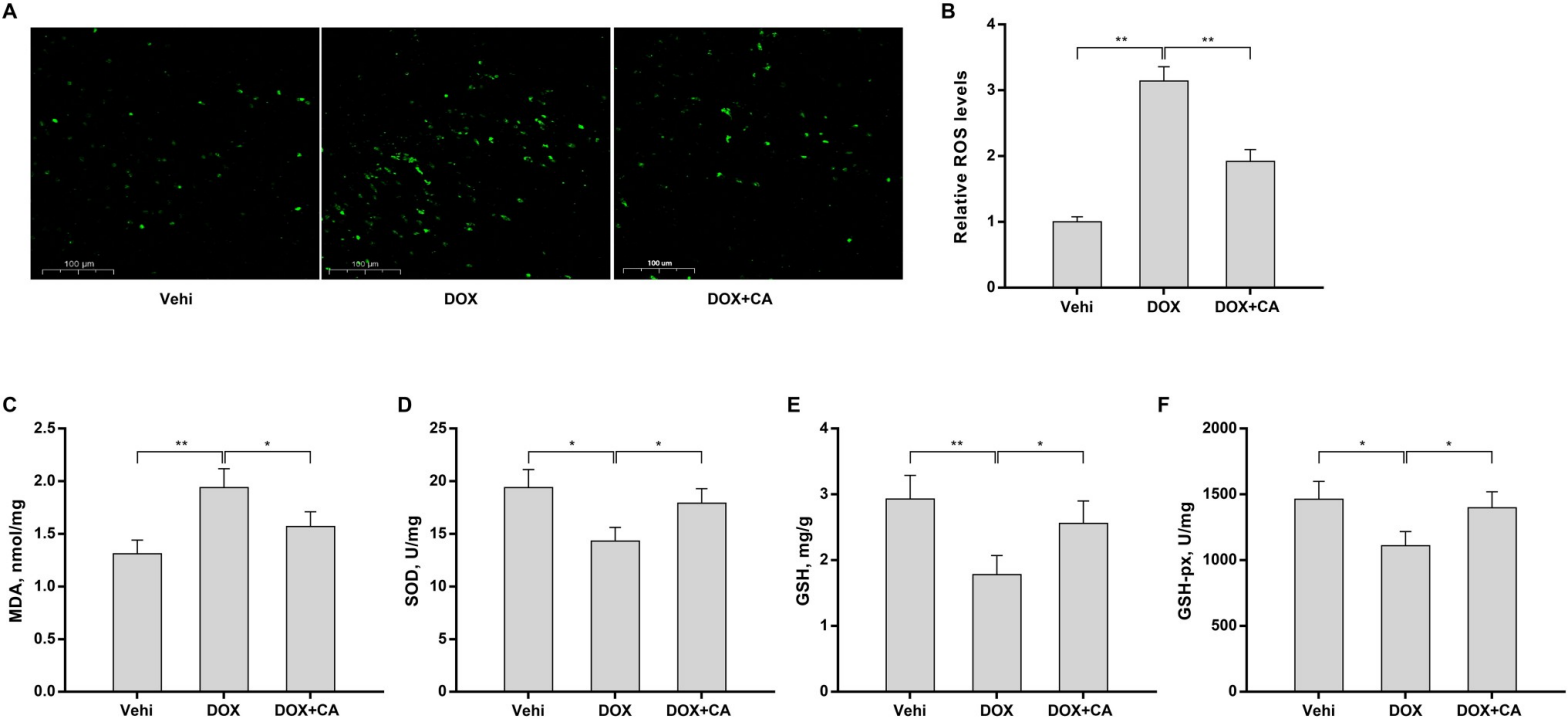
CA alleviated DOX-induced myocardial oxidative stress. (A and B) H9c2 cells were incubated with DOX (4 μM) and CA (100 μM), and intracellular ROS levels were measured using the DCFH-DA assay after 24 h (n = 3). MDA (C), SOD (D), GSH (E), and GSH-px (F) levels in cardiac tissues were assessed using commercial kits (n = 5). *p<0.05, **p<0.01.

Given that oxidative stress is a major mechanism of DOX-induced myocardial injury, we next investigated whether CA alleviated DOX-induced cardiotoxicity by decreasing oxidative stress. Fig 2A and 2B revealed that DOX obviously increased ROS levels in H9c2 cells, whereas CA repressed DOX-induced ROS. In a rat model of DOX-induced myocardial injury, DOX treatment suppressed SOD, GSH, and GSH-Px levels and increased MDA levels in cardiac tissues, whereas the effect was blocked by CA (Fig 2C–2F). These data suggest that CA effectively represses DOX-triggered myocardial oxidative stress *in vitro* and *in vivo*.

### CA activated Nrf2/HO-1 signaling

**Fig 3:**
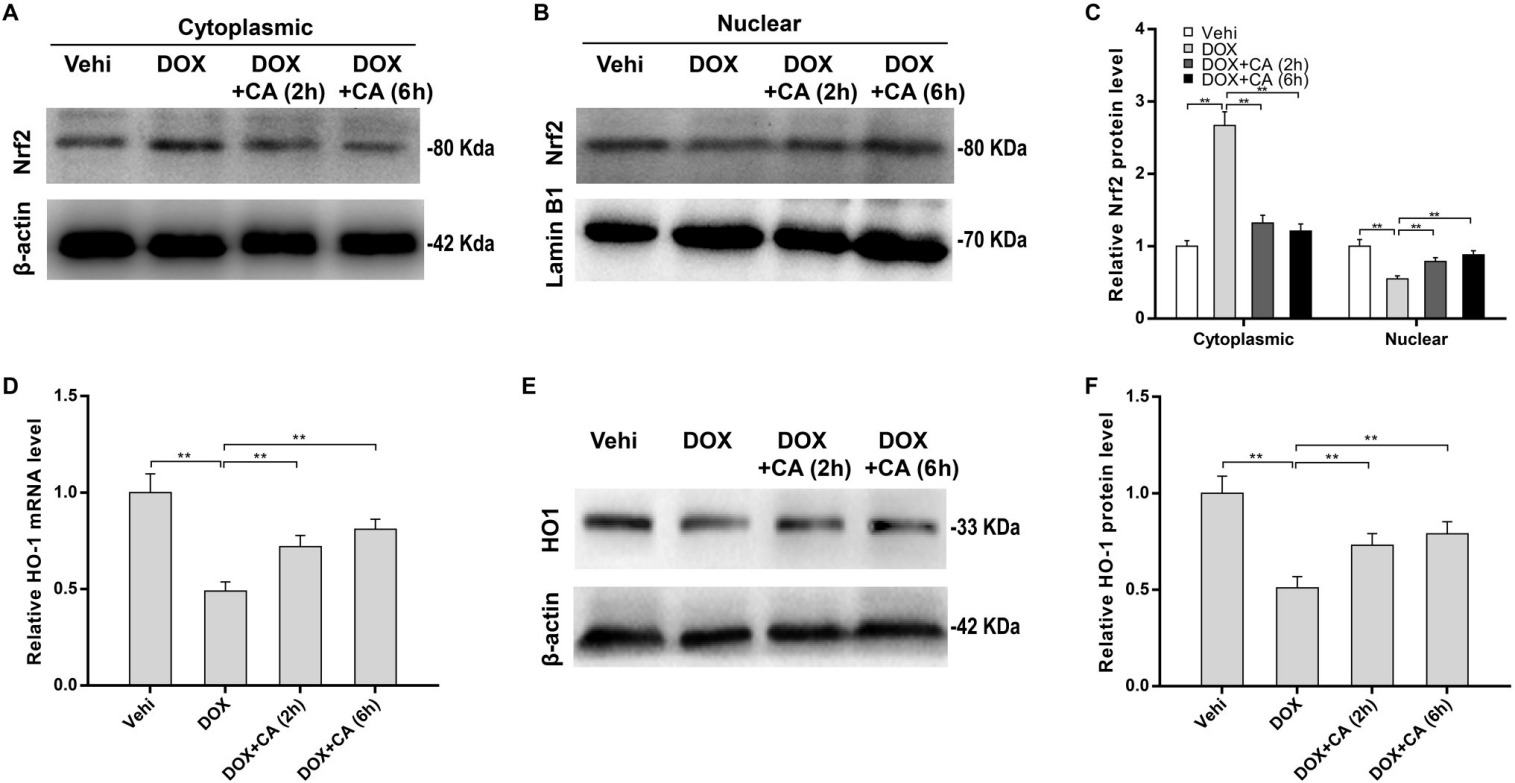
CA activated Nrf2/HO-1 signaling *in vitro*. Western blot assay (A) and quantification (C) of cytoplasmic Nrf2 protein levels in H9c2 cells treated with DOX (4 μM) and CA (100 μM) (n = 3). Western blot assay (B) and quantification (C) of nuclear Nrf2 protein levels in H9c2 cells treated with DOX (4 μM) and CA (100 μM) (n = 3). (D) qRT-PCR assay of HO-1 mRNA levels in H9c2 cells treated with DOX (4 μM) and CA (100 μM) (n = 3). Western blot assay (E) and quantification (F) of cytoplasmic HO-1 protein levels in H9c2 cells treated with DOX (4 μM) and CA (100 μM) (n = 3). **p<0.01.

**Fig 4:**
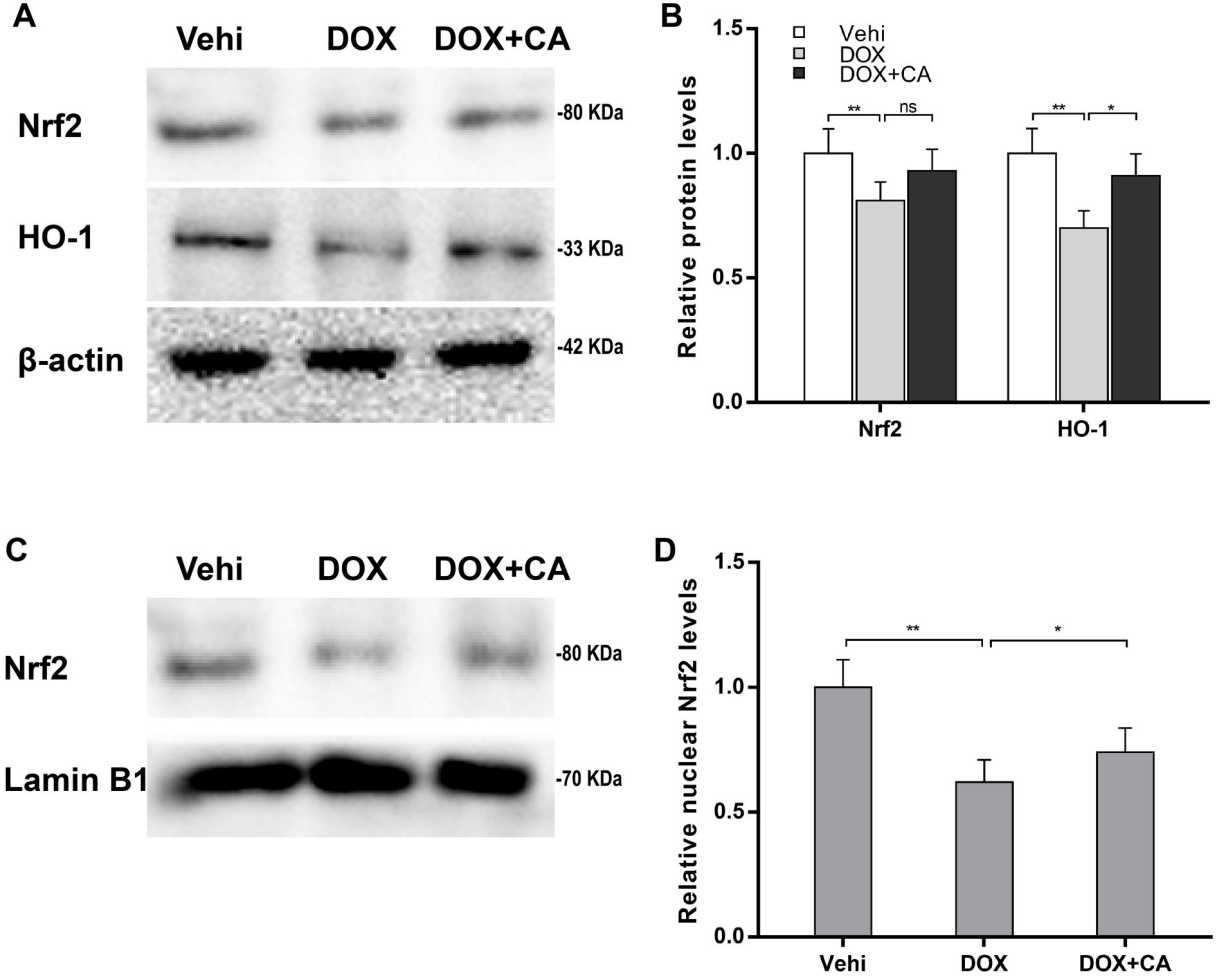
CA activated Nrf2/HO-1 signaling *in vivo*. (A and B) The total protein levels of Nrf2 and HO-1 in different groups of cardiac tissues (n = 5) were assessed using western blot assay. (C and D) The nuclear protein levels of Nrf2 in different groups of cardiac tissues (n = 5) were assessed using western blot assay.

Nrf2 exerts a critical role in the anti-oxidative defense by increasing anti-oxidant enzyme expression [37, 38], and CA possesses anti-oxidative capacity by promoting nuclear translocation of Nrf2 in human renal mesangial cells and dental pulp cells [28, 39]. Therefore, we further investigated whether CA alleviated myocardial oxidative stress by activating Nrf2/HO-1 signaling. Fig 3A–3C revealed that DOX markedly repressed nuclear translocation of Nrf2, as suggested by increased cytoplasmic Nrf2 levels and decreased nuclear Nrf2 levels. More importantly, Nrf2 was reactivated and tended to normalize after CA treatment (Fig 3A–3C). Then we investigated whether CA-induced Nrf2 activation further facilitated HO-1 expression, an Nrf2 target gene. Fig 3D–3F showed that DOX treatment suppressed HO-1 expression at the mRNA and protein levels in H9c2 cells, whereas the effect was blocked by CA in a time-dependent manner. The regulatory roles of CA in Nrf2/HO-1 signaling were further validated in animal models. Although CA did not restore the total Nrf2 levels in DOX-treated rats (Fig 4A and 4B), CA accelerated the nuclear translocation of Nrf2 (Fig 4C and 4D). Moreover, HO-1 expression was restored by CA in DOX-treated rats (Fig 4A and 4B).

### CA increased cardiomyocyte viability by repressing ferroptosis

**Fig 5:**
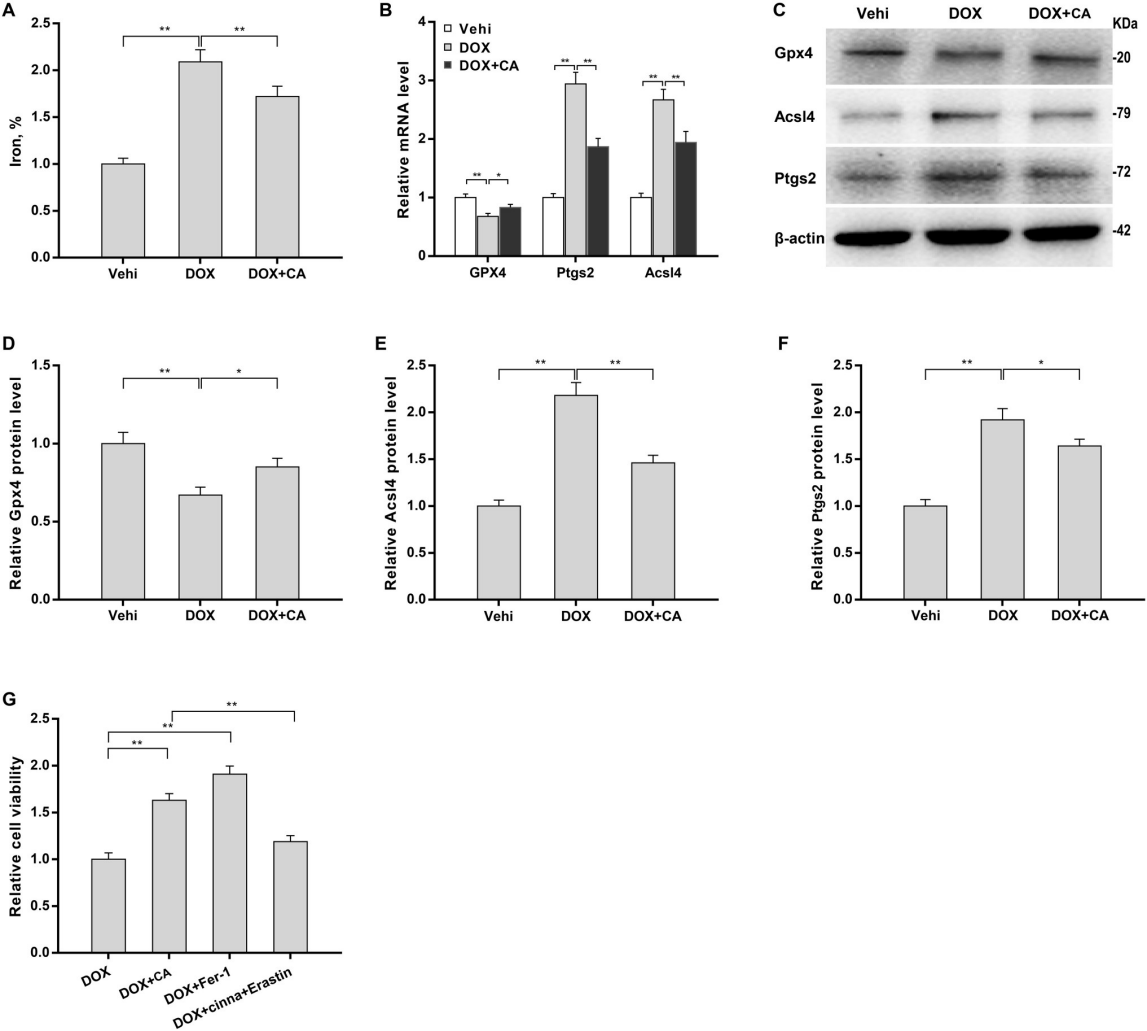
CA increased cardiomyocyte viability through repressing ferroptosis. (A) Relative iron concentration was assessed using commercial kits in H9c2 cells treated with DOX (4 μM) and CA (100 μM) (n = 3). (B) qRT-PCR assay of Gpx4, Ptgs2, and Acsl4 mRNA levels in H9c2 cells treated with DOX (4 μM) and CA (100 μM) (n = 3). Western blot assay (C) and quantification of Gpx4 (D), Acsl4 (E), and Ptgs2 (F) protein levels in H9c2 cells treated with DOX (4 μM) and CA (100 μM) (n = 3). (G) H9c2 cells were treated with 4 μM of DOX in the presence or absence of CA (100 μM), Fer-1 (1 μM), or Erastin (10 μM) for 24 h, and cell viability was measured with CCK8 (n = 3). *p<0.05, **p<0.01.

Nrf2 signaling is closely correlated with ferroptosis [40], which exerts an important role in myocardial oxidative injury [41, 42]. To assess the role of DOX and CA in ferroptosis, H9c2 cells were treated with DOX and CA, and then ferroptosis markers (iron concentration, ROS, MDA, and GSH levels) were examined. Fig 5A revealed that iron concentration was significantly increased in H9c2 cells after DOX treatment, whereas the effect was blocked by CA. The role of CA in repressing DOX-induced ferroptosis was also verified by assessing ROS, MDA, and GSH levels (Fig 2A, 2B and 2D). Furthermore, ferroptosis-related gene (Gpx4, Acsl4, and Ptgs2) levels were assessed in H9c2 cells after treatment with DOX and CA. Fig 5B showed DOX down-regulated Gpx4 mRNA levels and up-regulated Ptgs2 and Acsl4 mRNA levels, whereas CA reversed these effects. Western blot assay further revealed that DOX decreased Gpx4 protein expression and increased Ptgs2 and Acsl4 protein expression, whereas these effects were blocked by CA (Fig 5C–5F), indicating that CA repressed DOX-induced ferroptosis. Functionally, Fer-1 increased DOX-treated H9c2 cell viability like CA (Fig 5G). More importantly, Erastin, a selective ferroptosis activator, destroyed the effect of CA on restoring H9c2 cell viability (Fig 5G). These results demonstrate that ferroptosis is correlated with DOX-induced cardiotoxicity, and CA alleviates DOX-induced cardiotoxicity by inhibiting Nrf2-dependent ferroptosis.

## Discussion

Despite its efficacy against many malignancies, DOX exhibits severe cardiotoxicity, affecting approximately 30% of patients within five years after treatment [43]. Given that heart failure is a dominant reason for non-cancerous death after DOX treatment [43], relieving or eliminating DOX-triggered myocardial injury is essential to increasing its clinical application. Herein, we revealed the effect of CA on relieving DOX-induced cardiotoxicity. The main findings of this study were that: i) CA attenuated DOX-induced myocardial injury; ii) CA attenuated DOX-induced myocardial oxidative stress; iii) CA re-activated Nrf2/HO-1 signaling; and iv) CA enhanced cardiomyocyte viability by repressing cardiomyocyte ferroptosis.

Hydroxyl radicals, produced in physiological and pathological situations, are detoxified by GSH, which is a necessary antioxidant to maintain redox homeostasis through recycling enzymatic (GPXs, SOD, GST) and non-enzymatic (vitamin E) antioxidants [44]. DOX frequently results in GSH depletion through dysregulation of nicotinamide adenine dinucleotide phosphate (NADPH) [44]. NADPH is applied as the substrate of NADPH oxidases (NOXs) to generate ROS following DOX treatment [45]. Given that DOX-induced ROS overgeneration is a vital pathogenic event in cardiomyocyte injury [46], the roles of CA in alleviating DOX-induced oxidative stress and subsequent ROS-triggered cardiomyocyte ferroptosis were investigated. In the study, we demonstrated that DOX treatment increased ROS levels in H9c2 cells, whereas CA repressed DOX-induced ROS. Furthermore, DOX decreased SOD, GSH, and GSH-Px levels in the heart tissues of DOX-treated rats and increased MDA levels, whereas the effect was blocked by CA, indicating that CA effectively represses DOX-triggered myocardial oxidative stress *in vitro* and *in vivo*. CA further mitigated DOX-triggered cardiomyocyte ferroptosis. These results suggest that CA is a promising agent for decreasing the side effects of DOX by inhibiting ferroptosis.

Mounting studies have revealed the biological role of plant-derived natural compounds in regulating ROS production. Emodin, an anthraquinone extracted from rhubarb, exhibits a protective role against myocardial infarction through suppressing ROS generation [47]. Allicin sensitizes hepatocellular carcinoma (HCC) cells to 5-FU by further increasing ROS levels in cancer cells [48]. As an antioxidant, allicin relieves trastuzumab-induced cardiotoxicity by decreasing ROS-mediated myocardial cell apoptosis [49]. Astragaloside IV, an active component in Astragalus membranaceus, attenuates adriamycin-induced myocardial fibrosis through repressing cardiac ferroptosis and ROS levels [50].

The above results suggest that plant-derived natural ingredients possess the potential to relieve DOX-triggered myocardial injury by inhibiting DOX-induced oxidative stress. Given the biological role of CA in increasing Nrf2 nuclear translocation [27, 28] and repressing oxidative stress [28], we explored whether CA can alleviate DOX-induced cardiotoxicity through decreasing myocardial oxidative stress. The current results demonstrated that CA relieved DOX-induced myocardial oxidative stress by assessing ROS, MDA, SOD, and GSH levels. Mechanistically, CA accelerated the nuclear translocation of Nrf2 and increased HO-1 expression in cardiomyocytes. Furthermore, we found that CA repressed DOX-induced cardiomyocyte ferroptosis, while Erastin treatment destroyed the effect of CA on increasing cardiomyocyte viability. The major limitations of this study were that, i) besides ferroptosis, other ROS-triggered cell death forms (apoptosis, pyroptosis, and necrocytosis) were regulated by CA, and ii) it is essential to further investigate how Nrf2 signaling was activated by CA.

## Conclusions

These results suggest that CA alleviates DOX-induced cardiotoxicity, providing a promising opportunity to increase the clinical application of DOX.

## Supporting information

S1 Table qRT-PCR primers were used in the study. (DOCX)

S1 Raw images (PDF)
