# Supplementary material for: Cinnamaldehyde alleviates doxorubicin-induced cardiotoxicity by decreasing oxidative stress and ferroptosis in cardiomyocytes
Source: PLoS One. 2023 Oct 12;18(10):e0292124. doi: 10.1371/journal.pone.0292124 (PMC10569550; doi:10.1371/journal.pone.0292124)
Supplement: S1 Table — (DOCX) [file pone.0292124.s001.docx]

**Supplementary Table S1. qRT-PCR primers used in the study.**

| Genes | Sense (5’-3’) | Antisense (5’-3’) |
| --- | --- | --- |
| Col1α1 | TCCTTCTGGTCCTCGTGGTCT | CTTCCCCATCATCTCCGTTCT |
| α-SMA | GGCATCCACGAAACCACCTAT | CTCCTTCTGCATCCTGTCAGC |
| HO-1 | GAAGGCTTTAAGCTGGTGATGG | GTGGCTGGTGTGTAAGGGATG |
| Gpx4 | ATACGCCGAGTGTGGTTTACG | CCCTTGGGCTGGACTTTCAT |
| Acsl4 | AAGTCCATATCGCTCTGTCACG | TTTCTTCGCTCAGGATCTCCC |
| Ptgs2 | GATCTACCCTCCCCACGTCC | ACACTCTGTTGTGCTCCCGAA |
| β-actin | AAATCTGGCACCACACCTTCTAC | CAACATGATCTGGGTCATCTTCTC |
