# Supplementary material for: Cinnamaldehyde alleviates doxorubicin-induced cardiotoxicity by decreasing oxidative stress and ferroptosis in cardiomyocytes
Source: PLoS One. 2023 Oct 12;18(10):e0292124. doi: 10.1371/journal.pone.0292124 (PMC10569550; doi:10.1371/journal.pone.0292124)

Figure 3A-Nrf2

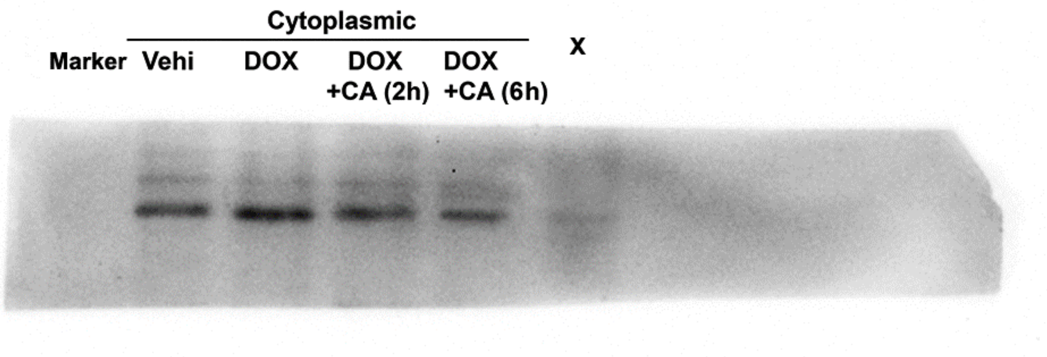

Figure 3A-Actin

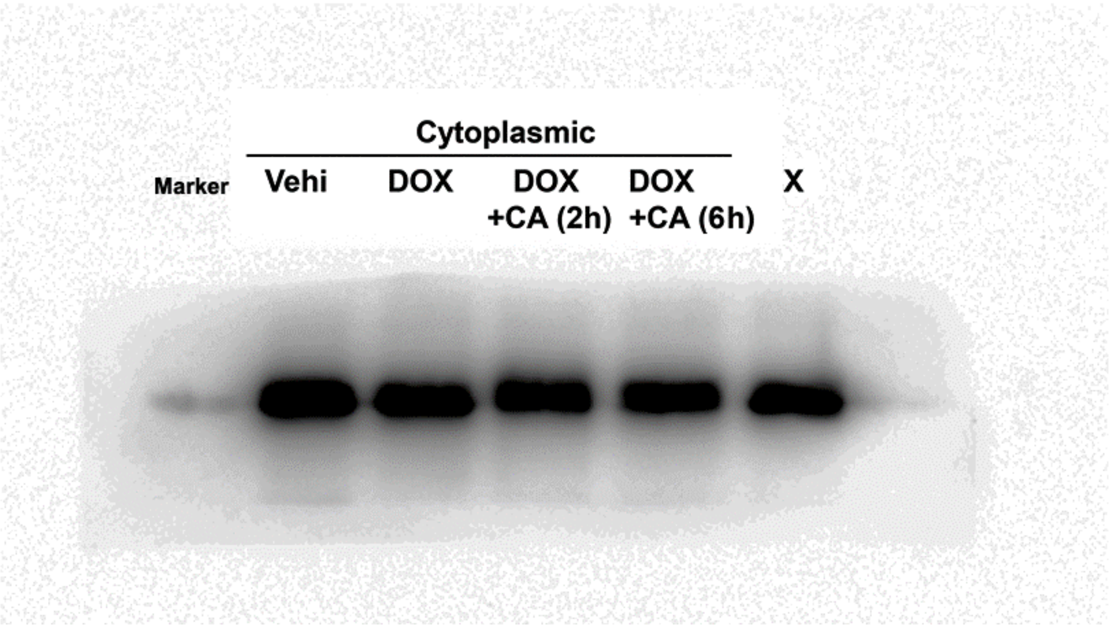

Figure 3B-Nrf2

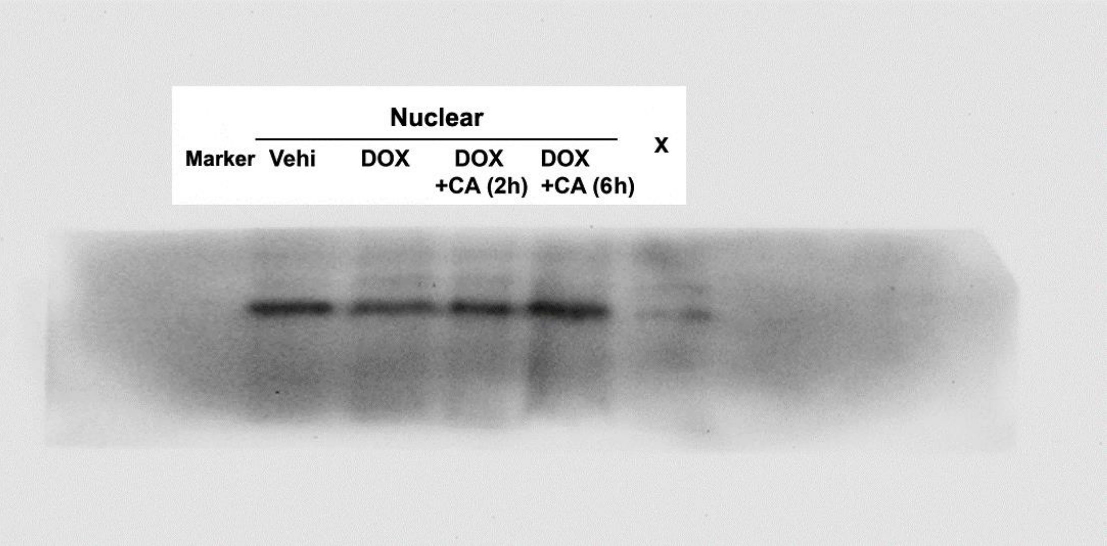

Figure 3B-Lamin B1

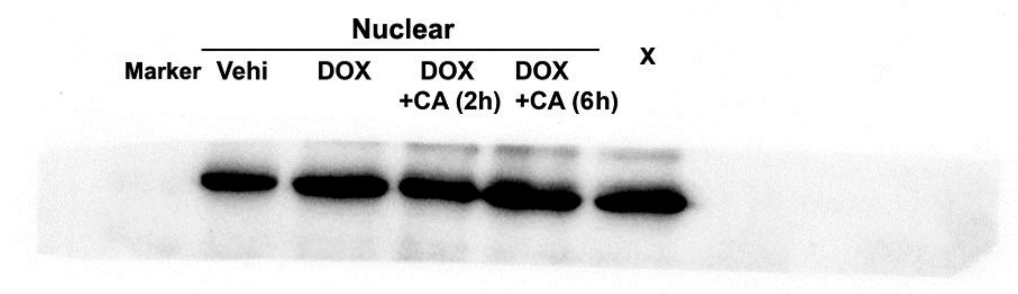

Figure 3E-HO-1

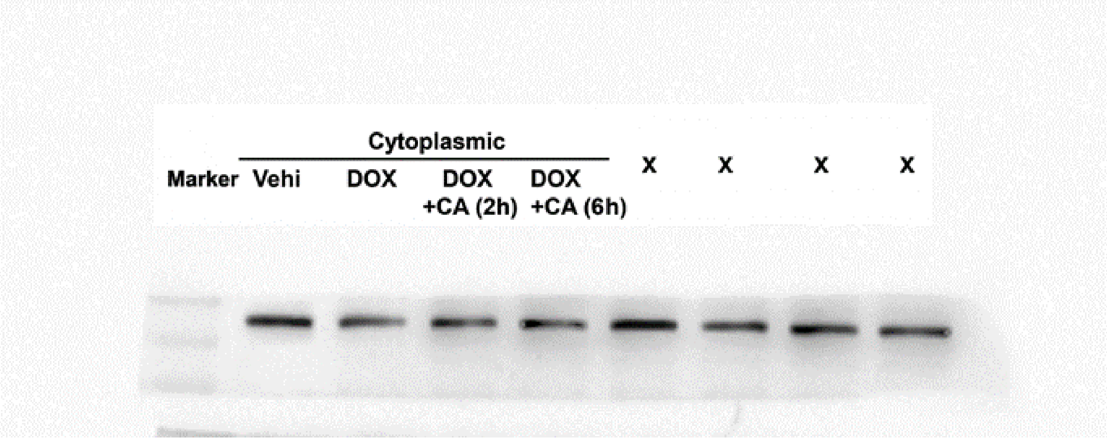

Figure 3E-ACTIN

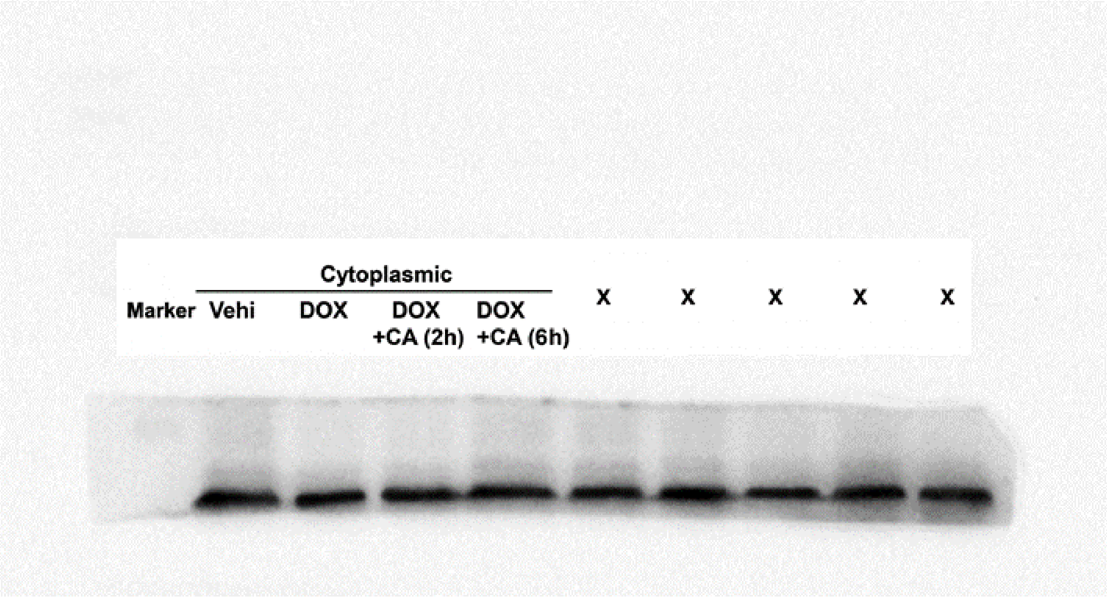

Figure 4 A and C---Nrf2

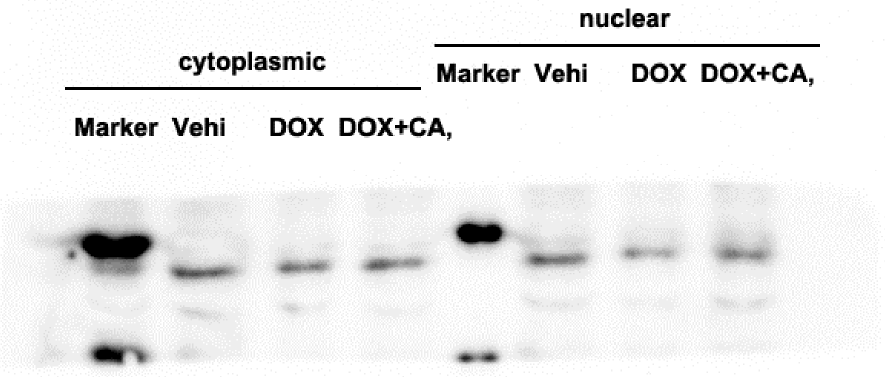

Figure 4 A---HO-1

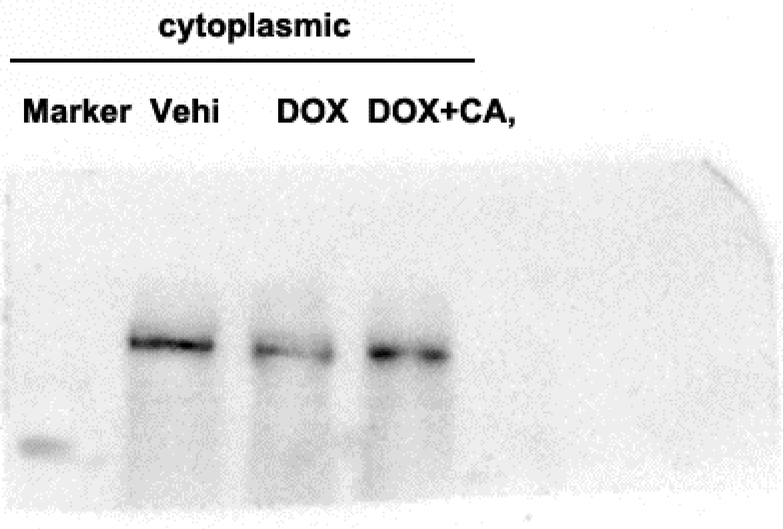

Figure 4 A---ACTIN

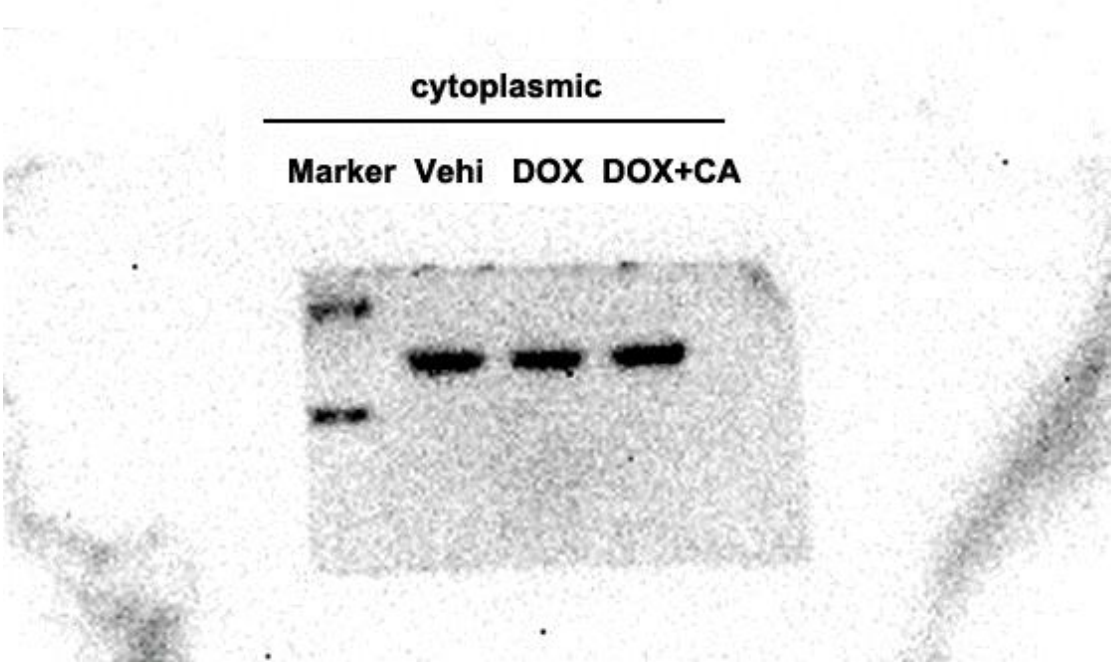

Figure 4 C---Lamin B1

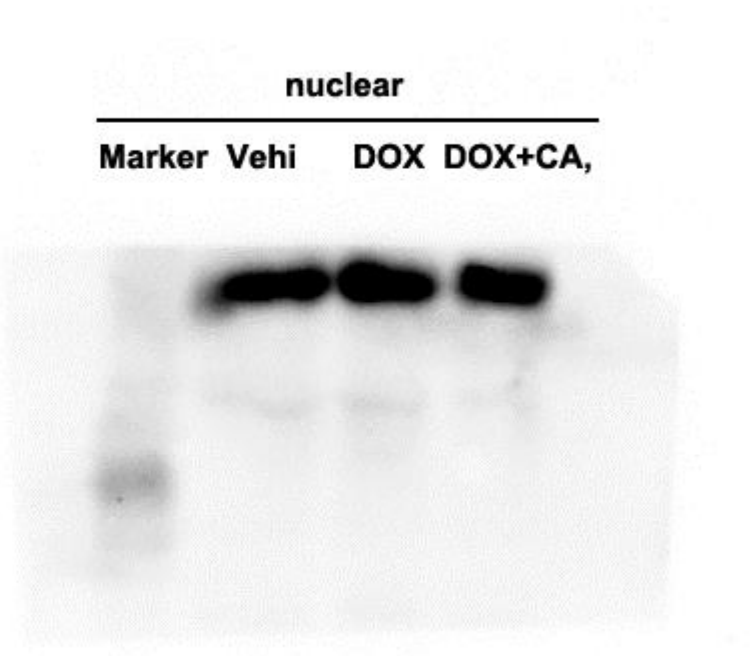

Figure 5C-Gpx4

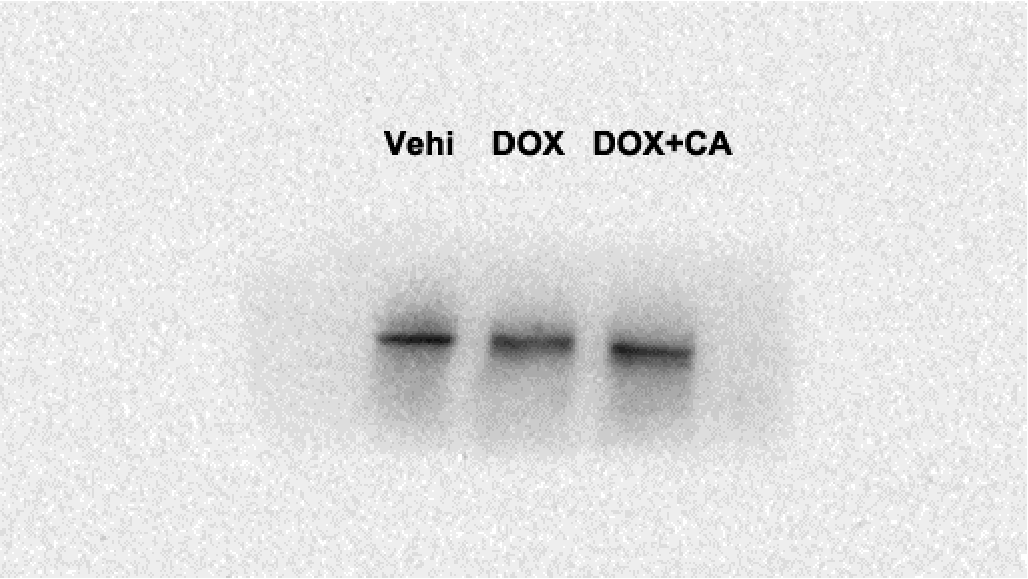

Figure 5C-Acsl4

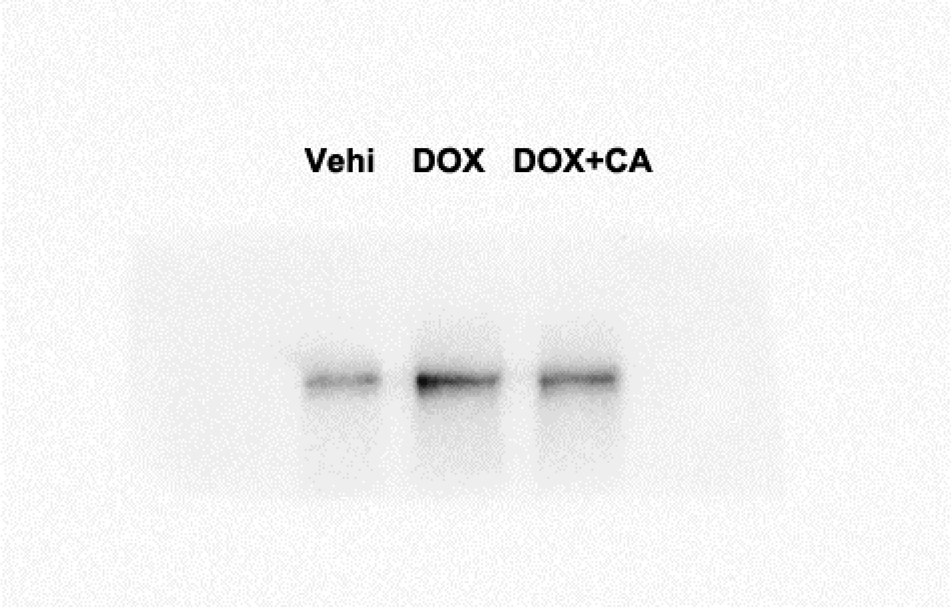

Figure 5C-Ptgs2

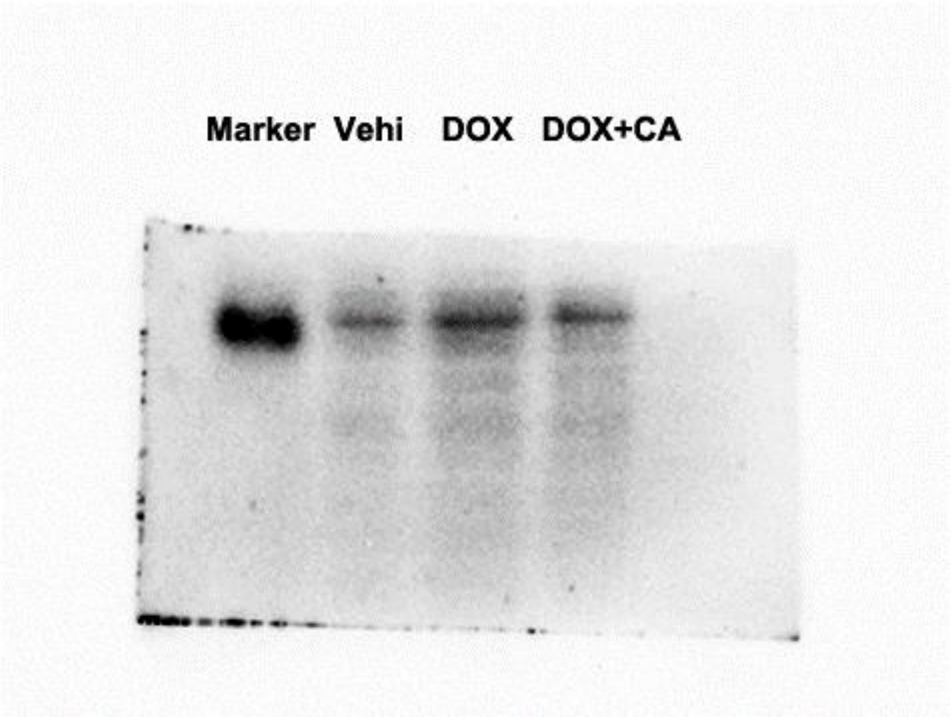

Figure 5C-Actin

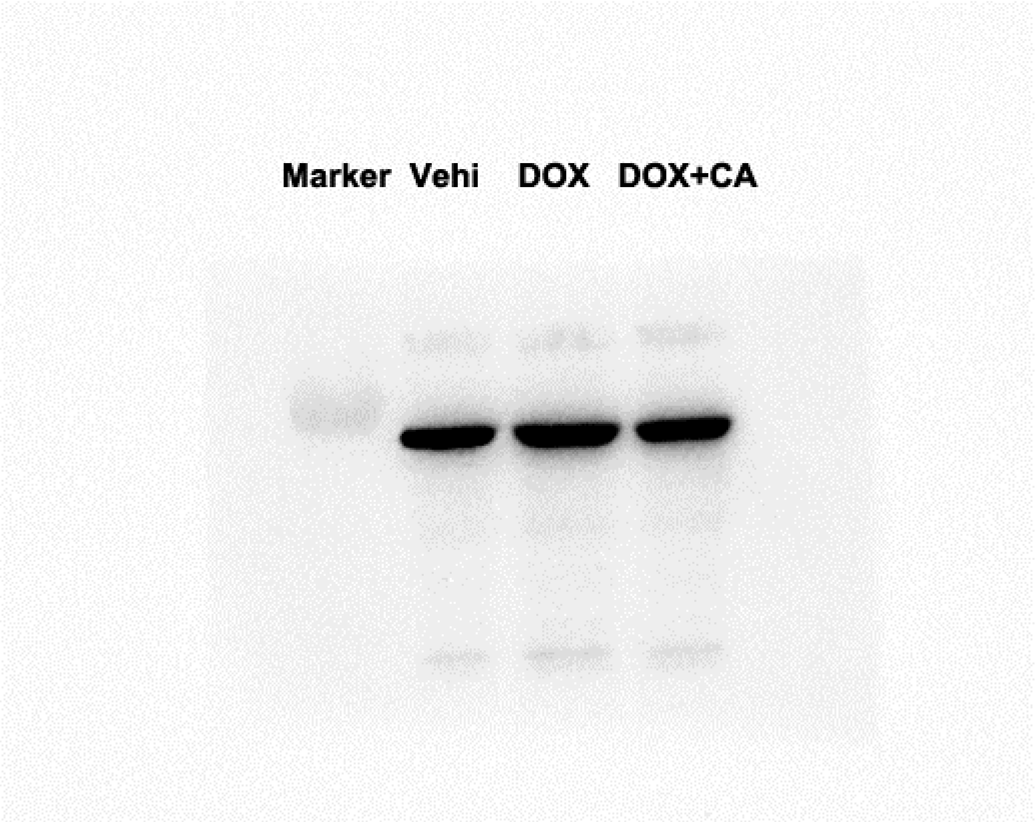

Supplement: S1 Raw images — (PDF) [file pone.0292124.s002.pdf]
